# Rheological and thermal properties of suspensions of microcapsules containing phase change materials

**DOI:** 10.1007/s00396-018-4316-9

**Published:** 2018-04-04

**Authors:** Vinh Duy Cao, Carlos Salas-Bringas, Reidar Barfod Schüller, Anna M. Szczotok, Marianne Hiorth, Manuel Carmona, Juan F. Rodriguez, Anna-Lena Kjøniksen

**Affiliations:** 1 Faculty of Engineering, Østfold University College, N-1757 Halden, Norway; 2 Department of Mathematical Sciences and Technology, Norwegian University of Life Sciences, N-1432 Ås, Norway; 3 Faculty of Chemistry, Biotechnology and Food Science, Norwegian University of Life Sciences, N-1432 Ås, Norway; 4 Department of Chemical Engineering, University of Castilla – La Mancha, 13004 Ciudad Real, Spain; 5 School of Pharmacy, University of Oslo, N-0316 Oslo, Norway

**Keywords:** Microencapsulated phase change materials, Non-encapsulated phase change materials, Heat storage capacity, Thermal conductivity, Shear thinning behaviour, Time-dependent behaviour

## Abstract

The thermal and rheological properties of suspensions of microencapsulated phase change materials (MPCM) in glycerol were investigated. When the microcapsule concentration is raised, the heat storage capacity of the suspensions becomes higher and a slight decline in the thermal conductivity of the suspensions is observed. The temperature-dependent shear-thinning behaviour of the suspensions was found to be strongly affected by non-encapsulated phase change materials (PCM). Accordingly, the rheological properties of the MPCM suspensions could be described by the Cross model below the PCM melting point while a power law model best described the data above the PCM melting point. The MPCM suspensions are interesting for energy storage and heat transfer applications. However, the non-encapsulated PCM contributes to the agglomeration of the microcapsules, which can lead to higher pumping consumption and clogging of piping systems.

## Introduction

The increasing cost of energy for heating and cooling creates a demand for more energy efficient buildings. Thermal energy storage (TES) systems using phase change materials (PCM) can be used to conserve and save energy. PCM are efficient energy storing materials due to a high heat capacity and a high latent heat per unit volume makes [1–5]. The use of microencapsulated phase change materials (MPCM) is an efficient way to store thermal energy, and has mainly been used in energy storage systems and for heat transfer applications [6–12].

Microcapsules suspended in a fluid have a great potential for thermal energy storage and heat transfer fluid applications [7–12]. Such suspensions can solve the problem with low thermal conductivity of PCM, and improve the specific heat capacity of the fluid within the PCM melting temperature range [8–10]. The flow and heat transfer characteristics of the microcapsule suspensions, and the mechanical stability of microcapsules under high shear rates are important for the efficiency of the systems. The most utilized fluid for microcapsule suspensions is water [8–11]. Water has some obvious advantages, including high thermal conductivity and a large specific heat capacity. In addition, it is cheap and available. Previous studies on water-based microcapsule suspensions have mainly examined the effect of microcapsule concentration and temperature on the thermal performance and rheological properties of the suspensions. It has been shown that the thermal conductivity and the specific heat capacity decrease when the concentration is raised. The suspensions exhibit a Newtonian fluid behaviour at low concentrations and pseudoplastic behaviour at high concentrations, while the relative viscosity of the suspensions is temperature independent [9–12].

One of the main problems of water-based MPCM suspensions is the high rate of microcapsule floatation. To solve this problem, a small amount of surfactants or thickeners can be used for improving the stability of suspensions [9]. Another solution is to utilize a fluid with a higher viscosity, such as glycerol. Glycerol has higher viscosity than water, thereby providing more stable suspensions. Furthermore, glycerol has lower freezing point and higher boiling point than water [13]. Accordingly, glycerol with high thermal conductivity and large specific heat capacity is a very interesting alternative to water as a carrier fluid for microcapsule suspensions intended for applications as thermal energy storage and heat transfer media.

Another problem with MPCM suspension is the formation of agglomerates. Agglomeration is unwanted for applications as heat transfer fluids due to an increase in viscosity, which causes a higher power consumption for pumping. In addition, the agglomerates may lead to clogging of piping systems [14].

In this article, suspensions of microcapsules in glycerol were investigated. The microcapsules are composed of a shell of low-density polyethylene (LDPE) and ethylvinylacetate (EVA) copolymers, and a core of paraffin Rubitherm®RT27, abbreviated LDPE-EVA/RT27. The LDPE-EVA/RT27 is suitable for TES applications due to the high latent heat (100 J/g), a melting point around 27 °C and the lack of interactions with the surrounding environment [15]. The effects of the microcapsules on the thermal performance and the rheological properties were investigated.

## Materials and methods

The microencapsulated phase change materials (MPCM) were made by a spray drying process [15]. The MPCM is composed of a paraffin Rubitherm®RT27 core coated with the LDPE-EVA (low-density polyethylene (LDPE) and ethylvinylacetate (EVA) copolymer) shell [15]. The surface morphology and the structure of the microcapsules were obtained by scanning electron microscopy (SEM) (FEI Quanta 200). The microcapsules size distribution was determined by laser light diffraction using a Malvern MasterSizer (Malvern Instruments Ltd., Malvern, Worcester, UK).

Suspensions of microencapsulated phase change materials were fabricated by dispensing different mass ratios of MPCM in glycerol at a room temperature. The mass concentration was varied from 0 to 30 wt.%.

### Thermal properties of MPCM suspensions

A Mettler Toledo DSC822e fitted with a MultiSTAR HSS7 sensor, under an inert atmosphere at a heating rate of 5 °C/min and a Hot Disk Instrument TPS 2500S at a heating power of 20 mW were employed to determine the thermal properties of MPCM suspensions. The latent heat of MPCM suspensions were determined by DSC while The Hot Disk Instrument was utilized to evaluate the thermal conductivity and volumetric heat capacity of the MPCM suspensions at room temperature (≈ 20 °C). Finally, thermogravimetric analyses (TGA) (TA Instrument equipment model SDT Q600) was used to determine the thermal stability of MPCM.

### Flow behaviour of MPCM suspensions

Rheological measurements were carried out using an Anton Paar MCR301 rheometer (Austria). The MPCM suspensions were tested using a CC27 bob/cop measuring system (cup diameter, 28.91 mm; bob diameter, 26.66 mm) mounted in a cylindrical Peltier for temperature control. A fresh sample was loaded into the measuring system. The sample was pre-sheared at shear rate of 50 s^−1^ for 5 min and rested for 5 min before any measurements were conducted. In order to investigate the reproducibility of the results, each measurement was repeated three times with fresh samples.

Flow curves were measured with a shear rate in the range of 10–500-10 s^−1^ at 10 °C, 20 °C (below the melting point of paraffin Rubitherm®RT27) and 40 °C, 50 °C (above the melting point of paraffin Rubitherm®RT27). The test was not performed at 30 °C to avoid the transition temperature of the melting process. The experimental data for the increasing shear rate curves were described by the Cross model (Eq.1) and the power law model (Eq. 2) [16]. The hysteresis areas between the increasing and decreasing shear rate curves were obtained using OriginPro 2016 Sr2.

The Cross model is usually used to describe the viscosity over a wide range of shear rates. The Cross model describes the suspension as a Newtonian fluid at low shear rates, and as a power law fluid at high shear rates:

1 $$ \eta ={\eta}_{\infty }+\left({\eta}_0-{\eta}_{\infty}\right){\left(1+{\left(\frac{\dot{\gamma}}{{\dot{\gamma}}_0}\right)}^2\right)}^{\frac{n-1}{2}} $$

where *η*_∞_, *η*_0_, and *n* are the viscosity at an infinite shear rate, the zero shear rate viscosity and the dimensionless flow behaviour index, respectively. $\dot{\gamma}$ and ${\dot{\gamma}}_0$ are the shear rate and the critical shear rate where the fluid transits from Newtonian to power law behaviour, respectively. In order to avoid unreliable data due to over-parameterization of the fitting procedure, the number of fitting parameters was reduced by subtracting the temperature-dependent viscosity of glycerol from the measured viscosity values. The resulting reduced viscosity values were then fitted to Eq. 1, fixing *η*_∞_ at zero. Although there are some deviation between the Cross model and the experimental data at low shear rates, the model gives a reasonably good fit to the data below the phase transition temperature of the paraffin core. Above the melting temperature of the paraffin core, the curves did not exhibit a Newtonian region in the considered shear rate range. Accordingly, at high temperatures Eq. 1 includes too many fitting parameters to achieve good fit of the data, and a simple power law behaviour (Eq. 2) was therefore used instead:

2 $$ \eta =\mathrm{K}{\dot{\upgamma}}^{n-1} $$

where *K* is the consistency index.

## Results and discussion

### Size distribution

**Fig. 1:**
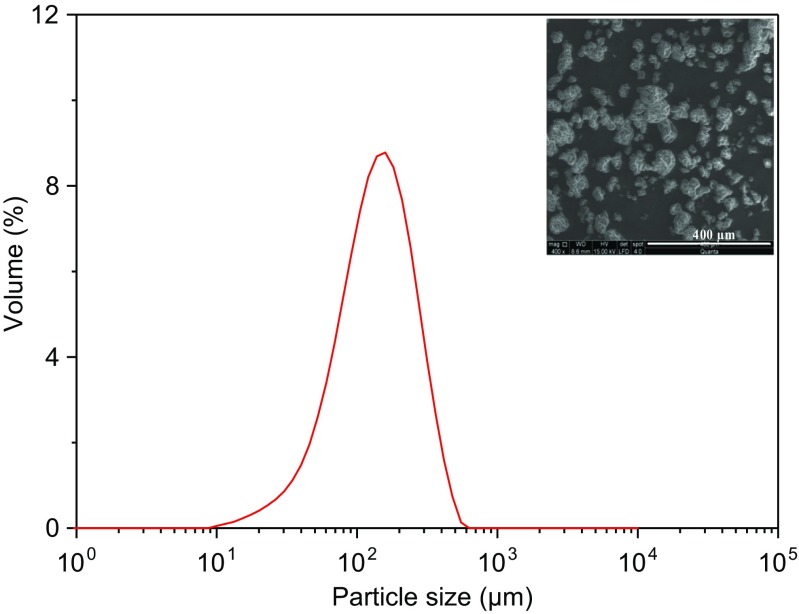
The size (diameter) distribution and inserted SEM image of the microcapsules

Before examining the rheological properties of the MPCM suspensions, it is important to know the size distribution of the microcapsules. Figure 1 illustrates that the particle size distribution (PSD) of the microcapsules are in the range of 10–550 μm with a median value of 170 μm (50% in the cumulative distribution). The inset plot in Fig. 1 shows a SEM image of the microcapsules, where the diameters of the single microcapsules is found to be about 3–10 μm. The much larger sizes observed in the particle size distribution indicates that the microcapsules form agglomerated structures.

### Thermal properties

**Fig. 2:**
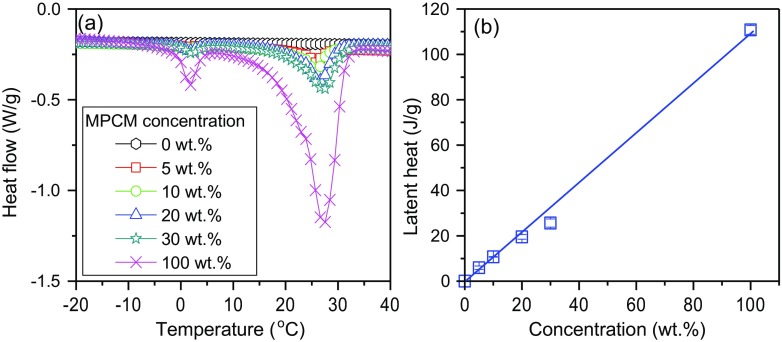
**a** The specific heat capacity and **b** the latent heat of the MPCM suspensions at different concentrations of microcapsules

All MPCM samples exhibited two distinct DSC peaks (Fig. 2a). The main peak represents the melting range temperature of the paraffin Rubitherm®RT27 core. The minor peak (0–5 °C) to the left of the main peak corresponds to the melting of water, which is present in the supplied Rubitherm®RT27. As can be seen from Fig. 2b, the latent heat of the suspensions is directly proportional to the MPCM concentration, confirming that the main peak is due to the melting of the paraffin. Addition of 30 wt.% of MPCM gives a latent heat of approximately 27 J/g.

**Fig. 3:**
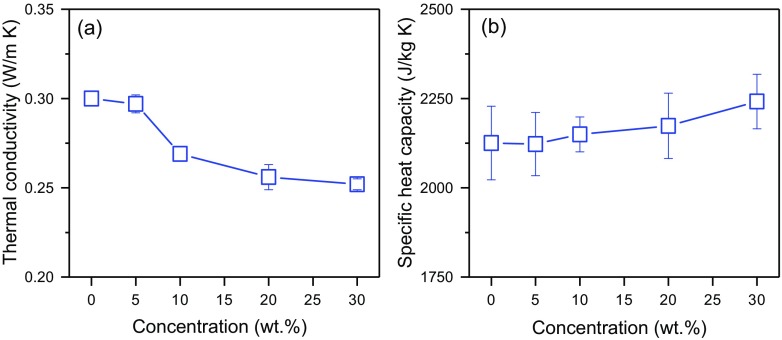
**a** The thermal conductivity and **b** the specific heat capacity of MPCM suspensions

The thermal conductivity and the specific heat capacity of the MPCM suspensions were determined using the transient plane source method (TPS). The experimental error was estimated by comparing the experimental thermal conductivity and specific heat capacity with reference values of pure glycerol. Figure 3 shows that the experimental thermal conductivity and specific heat capacity of pure glycerol are 0.300 ± 0.004 W/(m∙K) (reference value of 0.283 W/(m∙K) [17]) and 2126 ± 103 J/(kg∙K) (reference value of 2323 J/(kg∙K) [17]), respectively. Accordingly, the experimental errors of the method are about 6% for the thermal conductivity and 9% for the specific heat capacity.

Figure 3 shows the effect of MPCM addition on the thermal conductivity and specific heat capacity of the MPCM suspensions. The addition of MPCM causes a reduction of the thermal conductivity of the microcapsule suspensions (Fig. 3a). This is due to the lower thermal conductivity of the microcapsules compared to glycerol. The thermal conductivity of the paraffin Rubitherm®RT27 and polymer LDPE-EVA shell are approximately 0.2 W/(m∙K) and 0.13–0.34 W/(m∙K) [15], respectively. The thermal conductivity of glycerol is approximately 0.283 W/(m∙K) [17]. The specific heat capacity of the MPCM suspensions increases slightly when the concentration of MPCM is raised (Fig. 3b). This is due to the higher specific heat capacity of microcapsules at 20 °C compared to that of glycerol [17, 18].

**Fig. 4:**
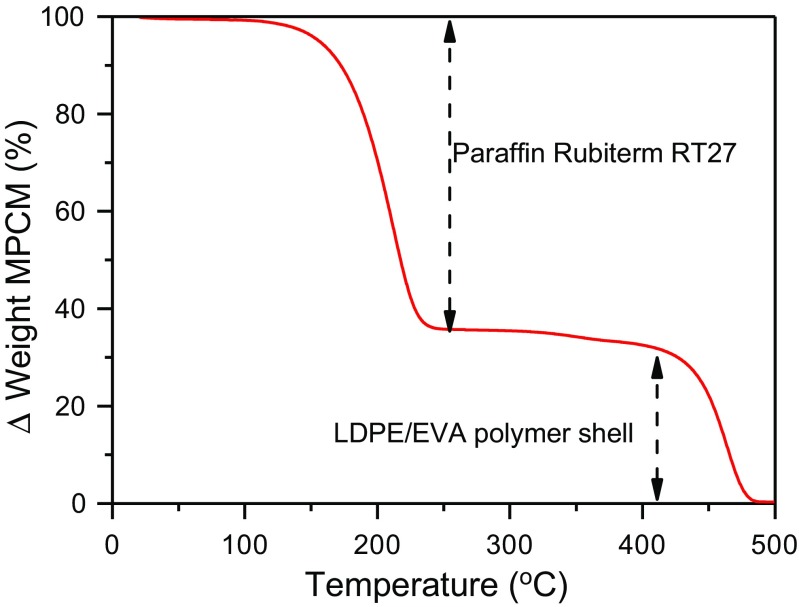
Thermogravimetric curve of the microcapsules

The thermogravimetric analysis of the microcapsules is shown in Fig. 4. There are two regions of weight loss in the thermal curve. The first region is between 150 and 250 °C, and is attributed to the evaporation of the paraffin Rubitherm®RT27. The second step is between 400 and 480 °C and is due to the degradation of the LDPE-EVA polymer shell [15, 19].

The thermal properties of the MPCM suspensions indicate that LDPE-EVA/RT27 is stable in the studied temperature range. Accordingly, the microcapsules are suited for integration into passive buildings, and the suspensions are interesting for heat transfer applications.

### Flow behaviour

**Fig. 5:**
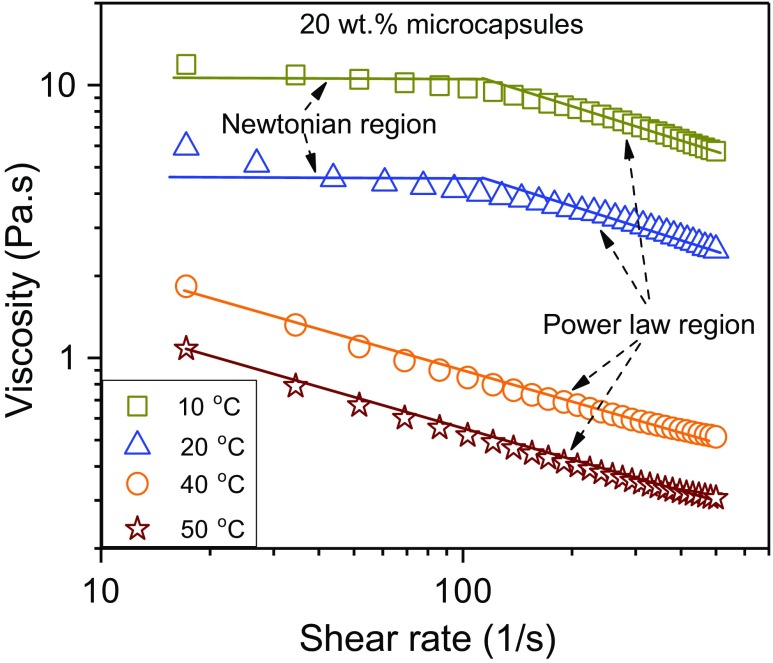
The effect of temperature and shear rate on the viscosity of 20 wt.% MPCM suspensions

Figure 5 shows the influence of temperature and shear rate on the viscosity of 20 wt.% microcapsule suspensions. The viscosity below the melting point of the paraffin core material (< 27 °C) exhibits a clear Newtonian region at low shear rates (10–100 s^−1^) followed by a power law region at high shear rates. However, above the melting point of paraffin, the Newtonian region is not reached within the considered shear rate range, and only the power law region is observed. Therefore, the flow curve of the MPCM suspensions was fitted to the Cross model (Eq. 1) below the melting point of paraffin, while the power law model (Eq. 2) was employed above the melting point.

**Fig. 6:**
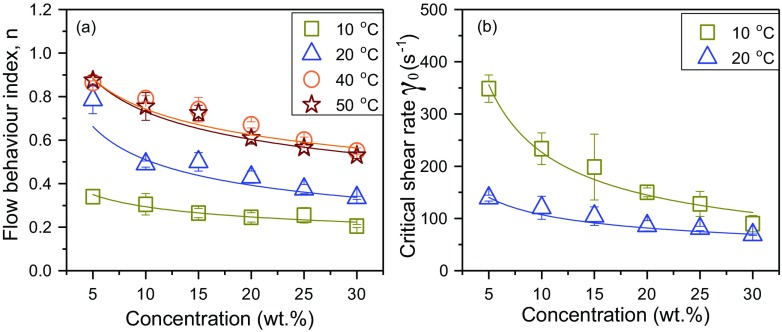
**a** The flow behaviour index *n* and **b** the critical shear rate ${\dot{\gamma}}_{\mathrm{o}}$ from power law and Cross models as a function of MPCM concentration and temperature

Figure 6 shows the flow behaviour index (*n*) obtained by fitting the experimental data to the power law model (Eq. 2) (above the melting point) and the Cross model (Eq. 1) (below the melting point) as a function of concentration and temperature. High values of *R*^2^ (0.98–1 for the Cross model and 0.99–1 for the power law model) reveals that both models are suitable for describing the flow behaviour of the MPCM suspensions in the considered temperature regions. The flow behaviour index *n* is less than one for all MPCM suspensions (0.21–0.88), illustrating that the samples exhibit strong and moderate shear-thinning behaviour. If the suspended microcapsules were present as single unagglomerated particles, a Newtonian behaviour without any shear-thinning effects would be expected. Accordingly, the shear-thinning behaviour suggests the presence of agglomerates that are broken down by the shear forces.

The flow behaviour index, *n*, decreases when the concentration is raised. This indicates a stronger shear thinning at higher concentrations, where there are more agglomerates that can be broken down by the shear forces. This is consistent with previous studies of other suspensions [20, 21]. As can be seen from Fig. 6a, the flow behaviour index, *n*, increases as the temperature is raised up to 40 °C, after which the temperature dependency is very small. Figure 6b shows the critical shear rate ${\dot{\gamma}}_0$ for the MPCM suspensions below the melting point of paraffin. The critical shear rate ${\dot{\gamma}}_0$ was found to decrease when the concentration of microcapsules was raised. This indicates that the agglomerates start to break down at lower shear rates when the concentration is increased. This suggests that the larger agglomerates present at high concentrations can be easier broken down compared to the smaller agglomerates at lower concentrations [22]. The critical shear rate${\dot{\gamma}}_0$decreases when the temperature is increased, and at high temperatures, the critical shear rate ${\dot{\gamma}}_0$ is below the considered shear rate range. Interestingly, both *n* and ${\dot{\gamma}}_0$ seem to be correlated with the melting point of paraffin. Until all paraffin has melted, *n* increases with temperature, while it is temperature independent when the sample is heated further. Below the melting point, ${\dot{\gamma}}_0$ becomes smaller with increasing temperature, while no Newtonian region is observed in the considered shear rate range above the melting point of paraffin. If all PCM were encapsulated in the microcapsules, we would not expect a distinct transition of the properties of the MPCM suspensions at the melting temperature of paraffin. Accordingly, non-encapsulated paraffin is probably causing this transition. Non-encapsulated paraffin can also contribute to the observed agglomeration of the microcapsules. When the sample is heated, paraffin becomes softer, and the associative forces within the agglomerates are reduced. This leads to higher values of *n* (reduced shear thinning), and shifts ${\dot{\gamma}}_0$ towards lower values (less force is needed to break the agglomerates apart). After the paraffin has melted, the agglomerates are easily disrupted and can be broken apart even at low shear rates, which is why no Newtonian plateau is observed at high temperatures.

**Fig. 7:**
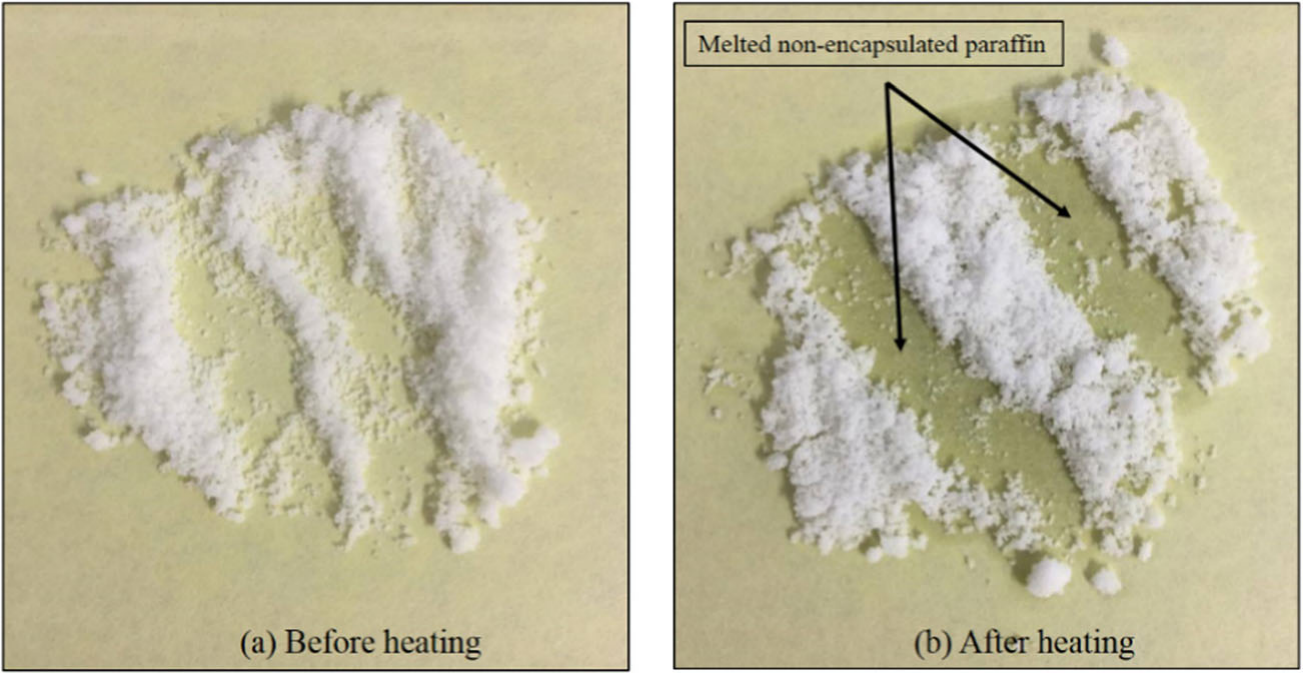
Images of an absorbing paper with (**a**) microcapsules before heating to 40 °C and **b** after heating to 40 °C for 10 min

Since the rheological data suggest the presence of non-encapsulated paraffin, an additional test was conducted to test this hypothesis. Microcapsules were weighed (0.5 g), and placed on an oil absorbing paper at room temperature. The paper was transferred to an oven at 40 °C for 10 min. Figure 7 shows the images of the absorbing paper with the microcapsules before and after the test. The change of colour and gloss of the paper indicates the presence of non-encapsulated paraffin. The amount of non-encapsulated paraffin was determined by weighting the absorbing paper before and after heating at 40 °C. According to this test, the MPCM contains approximately 2.5 wt.% non-encapsulated paraffin. Since the microcapsules utilized in this test were not subjected to disruptive forces, the non-encapsulated PCM is probably left from the synthesis process. The non-encapsulated paraffin will probably contribute to the observed agglomeration of the microcapsules. A disadvantage of free PCM is its tendency to clog distribution pipes for PCM heat transfer systems [23, 24].

**Fig. 8:**
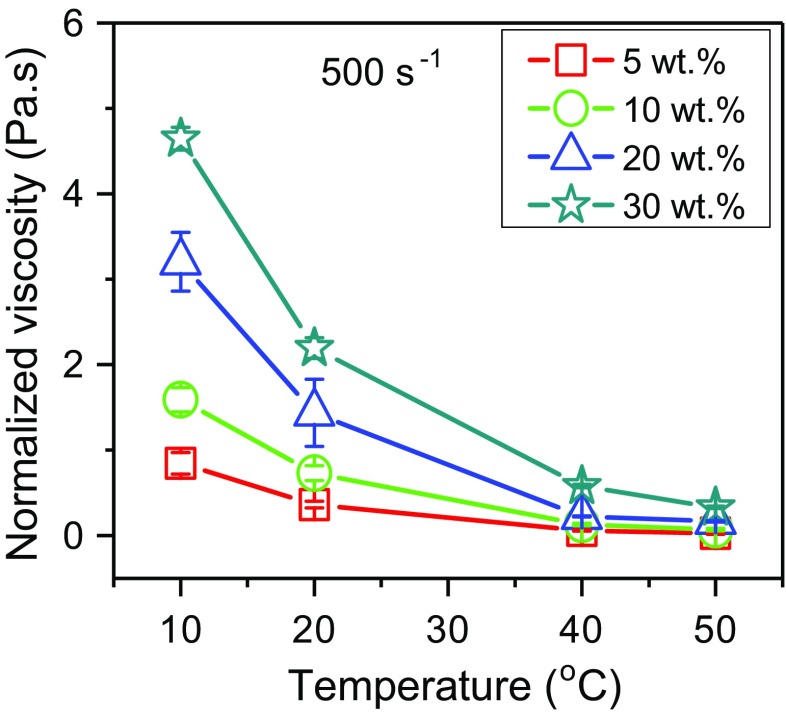
Effect of temperature on the viscosity measured at a shear rate of 500 s^−1^. Normalized by subtracting the viscosity of the solvent at the same temperatures

The normalized viscosity at 500 s^−1^ of the MPCM suspensions is plotted in Fig. 8. The viscosity of all MPCM suspensions decreases as the temperature is raised. The reduction of the normalized viscosity at elevated temperatures occurs because the kinetic energy of the microcapsules increases, leading to breakage of the inter-microcapsule bonds. This observation is supported by Nguyen et al. [25] and Fei Duan [26] studying water-based nanofluids. Furthermore, when the sample is heated, the non-encapsulated paraffin becomes softer, and the associative forces within the agglomerates are reduced, leading to a decrease in the viscosity of the MPCM suspensions.

**Fig. 9:**
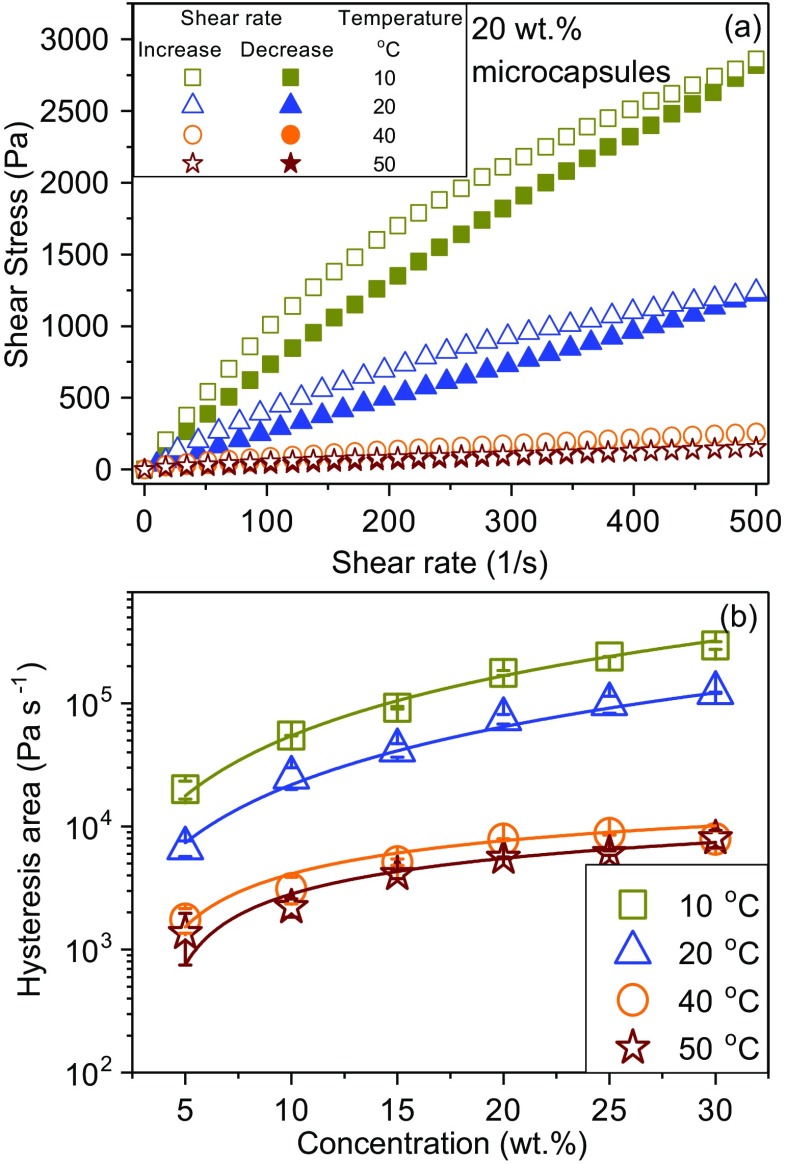
**a** Flow curves of the MPCM suspensions measured with increasing and decreasing shear rates at different temperatures at a MPCM concentration of 20 wt.%. **b** The hysteresis area of the MPCM suspensions as function of concentration and temperature

The flow curves of 20 wt.% of MPCM suspensions at different temperatures is shown in Fig. 9a. A hysteresis effect can be seen when the shear rate is increased and then decreased. When a sample is subjected to increasing shear rates followed by decreasing shear rates, the presence of a hysteresis area between the increasing curve and decreasing curve indicates that the flow of the sample is exhibiting a time-dependent behaviour [27]. The hysteresis effect suggests that the build-up of agglomerates when the shear rate is reduced is a slower process than the breakage of agglomerates at increasing shear rates. According to Roopa et al. [28] the loop area designates the energy required to break down the structure that is not recovered during the experimental period. The hysteresis area of the MPCM suspensions at different temperatures and concentrations are summarized in Fig. 9b.

Both the temperature and the concentration have a significant effect on the hysteresis loop area of the MPCM suspensions. The hysteresis area decreases as the temperature is increased from 10 to 50 °C at a constant concentration. The hysteresis area is much smaller at temperatures above the melting point of paraffin (40 and 50 °C) than for the lower temperatures. The hysteresis area of the MPCM suspensions are probably caused by the shear-induced break up of agglomerates, which need time to recover after being exposed to high shear forces [28]. Similar observations have been reported for hydrocolloid suspensions previously [29, 30]. Accordingly, the hysteresis effect is diminished when the non-encapsulated paraffin is melted, thereby reducing its effect on the agglomeration. The hysteresis area increases as the concentration is raised, which is probably due to the higher number of agglomerates in the sample.

## Conclusions

The rheological and thermal properties of suspensions of microcapsules containing phase change materials (MPCM) were investigated. The small diameter of single microcapsules (3–10 μm), the high latent heat (100 J/g) and high thermal stability (> 140 °C) of microcapsules are satisfactory for utilization in thermal energy storage and heat transfer applications. The thermal conductivity of the MPCM suspensions decreased with increasing MPCM concentration below the PCM melting point, while the specific heat capacity of the MPCM suspensions increased with the MPCM concentration. The latent heat of the MPCM suspensions increased to approximately 27 J/g by adding a 30 wt.% of MPCM, significantly improving the total heat storage capacity of the MPCM suspensions within the melting range of the phase change material (PCM).

Suspensions of microencapsulated phase change materials were found to exhibit shear-thinning behaviour. Interestingly, the rheological properties of the MPCM suspensions exhibited a transition around the melting temperature of the PCM. The presence of non-encapsulated PCM located outside the microcapsules was found to be the cause of this transition. The non-encapsulated PCM causes agglomeration of the microcapsules. Such structures will cause poor stability of MPCM suspensions and higher power consumption for pumping due to increased viscosity. When the PCM is melted, the binding force within the agglomerates becomes weaker. Accordingly, the hysteresis area of the flow curves decrease as the temperature is raised and it is significantly diminished above the PCM melting point. The Cross model was utilized to describe the rheological properties of the MPCM suspensions below the melting point of PCM, while a power law was used above the melting point due to the absence of a Newtonian region. The shear-thinning behaviour of the MPCM suspensions become stronger at higher MPCM concentrations and weaker at higher temperatures. The critical shear rate to break down the structure of the MPCM suspensions decreased when the temperature and concentration were increased.

Agglomerates may lead to clogging of piping systems. Improved microcapsules with reduced tendency for agglomerations and good mechanical properties would be interesting for further studies. In order to achieve such systems, it is important to avoid non-encapsulated PCM.
